# The Sun Healing Movement

**Published:** 1886-08

**Authors:** E. D. Babbitt


### *THE SUN-HEALING MOVEMENT.*

[From the *Herald of Health.*]

BY E. D. BABBITT, M.D.

The watchword of progress for to-day is "Come up to Nature; use all natural forces, such as water, earth, air, light, color." For many unhappy ages back, men have been too prone to go downward into the dark bowels of the earth after minerals; finally, they began to raise their eyes upward to the surface of the earth, and discovered something more refined and pure in the form of water and herbs, but a few sons of the morning raised their eyes heavenward, and awaking as from a dream, began to realize that the glorious sunlight is also a great promoter of health and life in the whole animal and vegetable world. This divine orb, resplendent in beauty, the greatest power in our solar system, has been staring men in the face all these thousands of years, trying to gain their attention so that they might receive its blessings; but loving darkness rather than light, they have kept on looking downward and dwelling among the grosser elements. Now, I would like to call out in thunder-tones to all my fellow-travelers and bid them waken! The sun is up! A new and brighter day is coming upon the earth!

Among a thousand things I would like to say on this subject, I must sift out only a few of the salient points in order to keep within the bounds of a magazine article.

In the first place, for the ordinary purposes of life, for all people who are well, or nearly well, the full white light as it comes from the sun is better than any of the colored rays, which are in themselves the constituent parts of that light. It is also most vitalizing and toughening to all feeble or negative persons. But the colored rays of sunlight as separated in a solar spectrum, or as strained off by means of colored glass, constitute all the different sides of power from warmth to coldness, consequently, in the treatment of disease, which is a one-sidedness of physiological action, we sometimes need some special color which develops the other side, and thus brings about the harmony which we call health. From the fact that physicians are generally ignorant of the chemistry and therapeutics of color, it may be best for me to dwell more on that department of the subject than upon the effect of light as a whole. Nevertheless, I will mere'y hint at some brief facts with reference to the full sunlight, reminding the reader that there are several times as much sickness on the shady side of a house or the northern slope of a mountain as upon the sunny side; that the insane idea of shading all windows, while saving the tints of the carpets slightly, will fade out human cheeks extensively; that there are patients whose cases have baffled the most eminent physicians while in shaded rooms, and yet they have been entirely healed by getting into the sunlight; that the nude nations of the world, who receive the light on their skin, are rarely, if ever, afflicted with scrofula or most of the other diseases, while their physical power is greater than that of the people who wear clothing: that the terrific calamities of idiocy, shocking deformities, goitres, etc., called cretinism, result from dwelling in the sunless valleys of the Alps; that living in cellars and shadowy places induces consumption, anemia with its ghastly pallor, shriveled features, depression of spirits, lifelessness, excitable nerves, tumors, ulcers, stupid mentality and many other perversions. "The only girls with rosy-red cheeks and sweet breaths," says Dio Lewis, "the only girls who become fully ripe and sweet, are those who baptize themselves freely in sunshine." And yet in railroad cars, horse cars and all other places where the blessed sunshine is trying to come in, our ladies usually shut it out in hot haste, for fear of having their complexions spoiled. It is true that in very hot weather it is best that the upper brain should be protected from the full sunlight, and also that a person walk on the shady side of the street; but in all moderately warm or in cold weather the sunny side is far better.

When the sun's rays are separated by a powerful prism, there are seen to be many grades of color-force arranged in an oblong form, which we call the solar spectrum, extending from the hot red end to the cool blue or violet end, and far beyond.

A trinity of the most distinctive colors is: Red and trans-red, or the center of heat; yellow, the center of light; blue, the center of cold and electricity.

The colors which act on the expansive plan, producing the effect of heat or thermism, are red, red-orange, orange, yellow-orange, yellow and yellow-green. Those which are contractive, working on the law of cold or electricity, are blue-green, blue, indigo-blue, indigo, violet-indigo, violet and dark violet.

I will have to be pardoned here in this brief article for presenting assertions without full proof, having given an abundance of facts to substantiate my points in my works on light and color.

Let me first state that each color is the sign of an exact style of force, and this is forever the same, whether it appears in the color of sunlight or the color of a hard substance, the only difference being that a colored ray of sunlight, like all fine forces, is more penetrating, safe, enduring and upbuilding to the mental system than the same color would be if connected with the ordinary coarser substances. Chromopathy, then, is really the best science of force as well as a system of color-cure. Let me state, also, that every color in a certain object tends to increase or stimulate the same color in another object. Now for a few brief proofs of these two laws.

Redness denotes something warm or burning, and also redness will increase the redness or heat of that with which it comes in contact. Thus red light will stimulate the arterial blood, which is itself red, with great power, and cause great heat, and red light will cause the thermometer to rise beyond any other color. Again, take capsicum, pink root, the oxides of iron, etc., all of which abound in redness, and they are known to be heating, "exciting to the arterial blood," "raising the pulse," etc. It is arterial blood, of course, which raises the pulse. In opposition to this it may be said that strawberries, tomatoes and some other red fruits are not heating in their nature. This is because the heating principle of red is balanced by acids, which are cooling. But the color-power of substances cannot always be told by their ordinary appearance, but must be revealed in the spectroscope. Thus, alcohol and ammonia have a strong burning and inflammatory principle, though not red externally. The leading element of both these substances, however, is hydrogen, which in the spectroscope gives a flaming red line. So potash is a white substance externally; but in the spectroscope potassium has its ruling lines in the red.

Let us go to the opposite extreme now, and notice the cold principle of blue. All acids in their legitimate action are cooling, and in the spectroscope have blue as a prominent color. The blue being cooling and contracting is, of course, anti-inflammatory, astringent, nervine, antiseptic, etc. Blue and violet light, in the same way, is quieting to all excited and overheated conditions, and the balancing principle is over-redness. But it would be absurd and hurtful to use blue light very much upon persons who are already pale and possessing blue veins, blue finger-nails and general coolness of temperament. Hence the absurdity of the one-idea system of blue light which prevailed some years ago, and which, though admirable to quell heat and excitability, is exactly wrong for one whose system is already too cold.

Again, the substance of the nerves has a predominance of yellow, with a certain amount of red, hence the yellow-orange is animating to the nerves. Yellow light over the bowels, for instance, will so animate them as to act as a laxative, or even a purgative, just as such yellow substances as colocynth, podophyllin, rhubarb, senna, sulphur, etc., taken internally will act in the same way. But I have another fact to reveal that my readers may consider rather startling, which is, that water and other substances upon which different colors of light may fall, become medicated until they have the same quality of power as the light itself. Thus many hundreds of cases have been cured of constipation by simply taking a common amber-colored bottle, which is nearly yellow-orange, filling it with water and letting it stand in the sun-light from half an hour upward, after which they drank one or two swallows at a time, especially before meals. One lady, whose system was almost a wreck, and who had tried everything else in vain, became bright, happy and strong by sipping yellow-charged water, and she called it *aqua vitæ*, the elixir of life. In rare cases a person may be found who from some special condition of the stomach, or from the food eaten, does not respond to its influence at once.

The blue-charged water of course acts as astringent, refrigerant and nervine, and has cured multitudes of cases of diarrhea, inflamed stomachs, summer complaints, etc. It is excellent for nervous persons to take a tablespoonful of it on retiring at night, and will often give them sweet sleep without any of the bad effects of poisonous narcotics. As a wash it is perhaps unequaled for softening the skin, curing chapped hands and relieving burns, hemorrage, weak eyes and all inflammatory conditions.

A word now about artificial lights. The opinion of a French engineer, quoted by the *Stientific American*, is that a yellow tint would be good to place around electric lights to relieve the eye; but it will be seen that according to the principles laid down above, this would be wholly unphysiological, the soothing colors being those which are cooling, such as blue; violet or even a cool green. Red lamp shades are a monstrosity. Dr. Forbes Winslow gives facts to show how injurious to the eyes are the red and yellow rays of the ordinary oil lamps and gas, and shows that a certain amount of blue in the lamp shades will prevent their bad effects. Pink and buff toned paper for newspapers are a great mistake. The best paper for the eyes is that which is nearly white, with a slight bluish or lilac tint. The toned paper of the future will be of that color, as it enables one to read with less irritation to the optic nerve.

It is time now that I had spoken of one great principle in nature's dynamics, namely, that fine forces reach down into the nervous and spiritual potencies of human systems, and, consequently, are more powerful and enduring in their effects than coarse elements. It is known that hot water drank on rising in the morning will relax the bowels; but they will not stay relaxed, and the dose must ever be repeated. Take, now, the more refined grade of heat that exists in yellow-charged water, and after a few times the bowels will be very likely to become so animated as to remain permanently cured, as I have proved by experment.

